# Assessment and Molecular Characterization of Human Intestinal Parasites in Bivalves from Orchard Beach, NY, USA

**DOI:** 10.3390/ijerph13040381

**Published:** 2016-03-29

**Authors:** Freda F. Tei, Steven Kowalyk, Jhenelle A. Reid, Matthew A. Presta, Rekha Yesudas, D.C. Ghislaine Mayer

**Affiliations:** Department of Biology, Manhattan College, 4513 Manhattan College Parkway, Riverdale, NY 10471, USA; ftei.student@manhattan.edu (F.F.T.); skowalyk.student@manhattan.edu (S.K.); jreid.student@manhattan.edu (J.A.R.); mpresta.student@manhattan.edu (M.A.P.); rekha.yesudas@manhattan.edu (R.Y.)

**Keywords:** bivalves, mussels, *Cryptosporidium*, *T. gondii*, Orchard Beach, bio-sentinels

## Abstract

Bivalves have been shown to be carriers of the human intestinal parasites *Cryptosporidium parvum* and *Toxoplasma gondii*. The goal of this study is to determine the prevalence of protozoan parasites in mollusks of New York City using a polymerase chain reaction (PCR)-based assay. Four species of mollusks, *Mya arenaria, Geukensia demissa, Crassostrea virginica*, and *Mytilis edulis*, were collected from Orchard Beach, NY in the fall of 2014, totaling 159 specimens. Each individual mollusk was dissected to harvest the digestive gland, the mantle, the gills, the foot and the siphon. The tissues were assayed for the presence of *Cryptosporidium parvum*, *Giardia lamblia*, and *Toxoplasma gondii* DNA by using primers that target parasite-specific genes. *C. parvum* was found at a prevalence of 50%, 11.3%, and 1%, respectively, in *Mya arenaria*, *G. demissa*, and *Mytilis edulis*. *C. parvum* DNA was detected in all the tissues of these bivalve species, except the gills. Furthermore, *G. lamblia* was detected in *Mya arenaria*, *G. demissa*, *Crassostrea virginica* and *Mytilis edulis* at a prevalence of 37.5%, 4.5%, 60%, and 20.6%, respectively, while *T. gondii* DNA was not detected.

## 1. Introduction

Bivalve mollusks are frequently used as bio-indicators in the appraisal of environmental quality. Among several features, their extensive geographic distribution and their stationary nature make them valuable indictors for chemical and biological pollution. As suspension-feeding organisms, they are exposed and accumulate considerable amount of pollutants, although those pollutants might be present at low concentration [1,2,3]. They have previously been used to monitor metal contamination of water [4,5].

Recent studies have found *Toxoplasma gondii* and *Cryptosporidium parvum*, two important human parasites, in several species of marine bivalves [6,7,8,9,10]. Thus, bivalves could be used as bio-sentinels for human parasites. According to the Center for Diseases Control (CDC), *Cryptosporidium* is the leading cause of water-borne disease among humans in the United States [11]. The mode of transmission for *Cryptosporidium* is through contaminated water and food or direct contact with an infected host. The oocysts have a thick shell that help the parasite to survive for long periods of time until it infects a definitive host.

Orchard beach is a public beach located in Pelham Bay Park in the Bronx borough of New York City. The goal of the present study is to determine the prevalence of human intestinal parasites in wild populations from Orchard Beach using molecular diagnostics tools.

## 2. Experimental Section

### 2.1. Collection and Sample Processing

**Figure 1:**
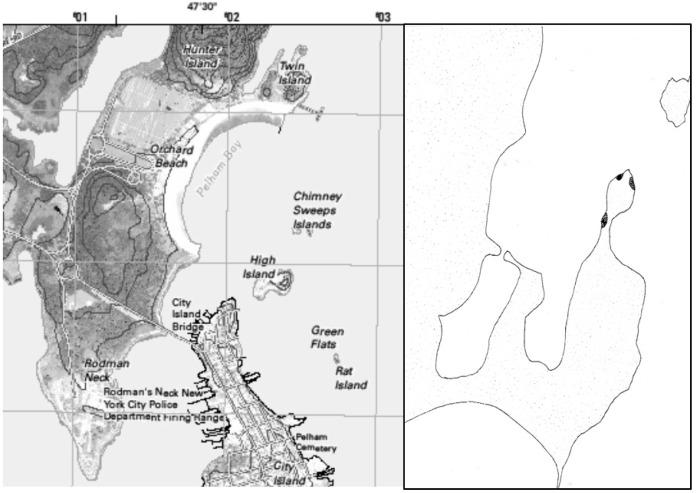
Map of the collection site. Modified from the United States Geological Survey.

Bivalves were collected on Orchard beach, a public beach located in the Bronx in New York City. Collection was restricted to the Twin Island section of the park (40.871186° N, 73.784389° W) (Figure 1). A total of 159 bivalves were collected at low tide on 9 September, 2014 belonging to four species. The bivalves collected were longneck steamers (*Mya arenaria*, 8), ribbed mussels (*Geukensia demissa*, 44) Atlantic oysters (*Crassostrea virginica*, 10), and blue mussels (*Mytilis edulis*, 97). They were all burrowed in the sand at the time of collection, except for *Crassostrea virginica*, which were attached to rocks. The samples were kept alive moist in sea water at 4 °C prior to dissection and DNA isolation. Following dissection, the tissues were stored at −80 °C.

### 2.2. DNA Extraction and Molecular Analysis

After thawing of the tissues, DNA was extracted from each tissue by using the DNAeasy Blood and Tissue extraction kit from Qiagen (Germantown, MD, USA). DNA purity was measured using the Nanodrop. The bivalves were tested for *C. parvum*, *Giardia lamblia*, and *Toxoplasma gondii*. All PCR assays were performed using 300 ng of purified DNA per reaction, PCR master mix (Qiagen, Germantown, MD, USA), parasite specific primers at 20 pmol/µL in a final volume of 25 µL. To detect *G. lamblia*, nested-PCR was performed using primers that target the β-giardin gene following a previously described protocol [12]. The forward primer for the first reaction was Gia7 (5′-AAGCCCGACGACCTCACCCGCAGTGC-3′) and the reverse primer was Gia759 (5′-GAGGCCGCCCTGGATCTTCGAGACGAC-3′). The nested PCR reaction was amplified using the forward primer (5′-GAACGAACGAGATCGAGGTCCG-3′) and the reverse primer (5′-CTCGACGAGCTT CGTGTT-3′). The genotype was assessed by digesting the amplicons with the restriction endonuclease *HaeIII*. Briefly, 20 µL of the total volume PCR reactions were digested with 10 unit of *HaeIII* for 4 h at 37 °C. To detect the presence of *C. parvum* DNA, PCR was performed using the LAX primer pairs previously described to specific for *C. parvum* [13,14]. The primer sequences are as follows forward: LAX469F 5′-CCGAGTTTGATCCAAAAAGTTACGAA-3′, and LAX869R.

5′-TAGCTCCTCATATGCCTTATTGAGTA-3′. The following conditions were used: 94 °C for 3 min, 94 °C for 45 s, 52 °C for 45 s, and 72 °C for 1 min, a final extension at 72 °C for 7 min. *C. parvum* purified DNA was used as a positive control [14]. *T. gondii* DNA was detected by using the *T. gondii* GRA6 primer sets (forward 5′-GTAGCGTGCTTGTTGGCGAC-3′, reverse primer, 5′-ACAAGACATAGAGTGCCCC-3′). The PCR reactions were performed as described by Fazaeli *et al.* [15]. The PCR products were detected by ultraviolet light using agarose gels stained with ethidium bromide. Ten nanograms of purified parasite DNA was used as a positive control and water was used as a negative control for all PCR reactions. The resulting PCR products were sequenced and the genotype was analyzed with Sequencher 5.3.

## 3. Results and Discussion

### 3.1. Prevalence of Human Intestinal Parasites in Bivalves from Orchard Beach

A total of 159 specimens of bivalves belonging to four species were collected at low tide in the fall of 2014 to test for exposure to human intestinal parasites. We found that specimens of *Mya arenaria* (4/8), *Geukensia demissa* (5/44) and *Mytilis edulis* (1/97) tested positive for *C. parvum* DNA, while all the specimens of the Atlantic oysters were negative for *C. parvum* DNA (Table 1). In total, 50% of the tested *Mya arenaria* were positive for *C. parvum*, while 11% of the tested *Geukensia demissa* and 1% of *Mytilis edulis* were infected with *C. parvum* DNA, respectively. Sequence analysis confirmed the presence of *C. parvum* DNA in all positive samples. In contrast, 60% of the tested *Crassostrea virginica* was positive for *G. lamblia* DNA, while 37.5% of *Mya arenaria*, and 4.5% of the *Geukensia demissa* were positive, respectively (Table 1). Our data indicate contamination of the water of Orchard beach with both *G. lamblia* and *C. parvum* oocysts. We did not detect *T. gondii* DNA in any of the specimens suggesting no contamination with *T. gondii* oocysts at Orchard beach.

### 3.2. Parasite DNA Tissue Distribution

We determined the tissue distribution of *C*. *parvum* and *G. lamblia* by dissecting the foot, mantle, digestive glands, gills, siphon and abductor muscle (*Crassostrea virginica* only) of each collected bivalve specimen. We found that parasite DNA was found predominantly in the gills and digestive glands (Table 2 and Table 3). Interestingly, *G. lamblia* DNA was not found in the mantle and digestive glands of *Geukensia demissa*. *C. parvum.* DNA was only detected in the foot and gills of *Mytilis edulis* (Table 2 and Table 3). Although the sample size for *Mya arenaria* consisted of eight specimens, we found co-infection of two specimens of *Mya arenaria* with *C. parvum* and *G. lamblia* (Table 2 and Table 3). The tissue distribution was distinct in the co-infected specimens. In specimen 4, *G. lamblia* DNA was detected in the siphon, while *C. parvum* DNA was detected in the foot and digestive glands (Table 2 and Table 3). In contrast, *G. lamblia* DNA was found in the mantle of specimen 5, while *C. parvum* DNA was detected in the foot (Table 2 and Table 3). Parasite DNA was not detected in every dissected tissues of the bivalve specimens, suggesting specific tissue distribution and little cross-contamination during the dissection (Table 2 and Table 3).

### 3.3. Genotyping of G. lamblia from Bivalves Collected at Orchard Beach

**Figure 2:**
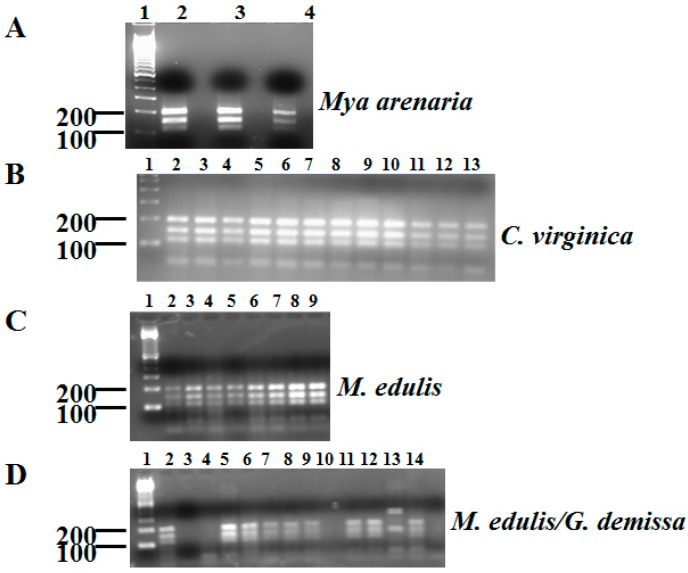
Genotyping of *G. lamblia* DNA from bivalves collected from Orchard beach, NY PCH-RFLP. (**A**) Genotyping of *G. lamblia* PCR products from *Mya arenaria* by digestion of secondary PCR with *HaeIII*, 50 bp fragment migrated out of the gel. Lane 1, 100 bp marker; lanes 2–6 *G. lanblia* assemblage A; (**B**) Genotyping of *G. lamblia* PCR products from *C. virginica* by digestion of secondary PCR with *HaeIII.* Lane 1, 100 bp marker; lanes 2–13 *G. lanblia* assemblage A; (**C**) Genotyping of *G. lamblia* PCR products from *M. edulis* by digestion of secondary PCR with *HaeIII.* Lane 1, 100 bp marker; lanes 2, *G. lanblia* assemblage A, lanes 3–4, *G. lanblia* assemblage C, lanes 6–9, *G. lanblia* assemblage A; (**D**) Genotyping of *G. lamblia* PCR products from *M. edulis* (lanes 2–12) and *G. demissa* (lanes 13–15) by digestion of secondary PCR with *HaeIII.* Lane 1, 100 bp marker; lanes 2, *G. lanblia* assemblage A, lanes 3-4 undetermined genotype, lanes 5–9 *G. lanblia* assemblage C, lanes 10 undetermined genotype, lanes 11-12 *G. lanblia* assemblage A, lanes 13, *G. lanblia* assemblage D, lanes 14, *G. lanblia* assemblage C, lanes 15, undetermined genotype.

Genetic variation is common among *G. lamblia* strains [16,17]. Seven genotypes are commonly accepted as distinctive evolutionary lineages by restriction fragment length polymorphism (RFLP) [18,19]. Several studies have revealed multiple differences among these lineages, including drug sensitivity, metabolism, and host preference [18,19]. Humans are only infected by assemblages A and B, while assemblages C and D infect dogs; hoofed animals are infected by assemblage E [18]. Moreover, assemblage F infects cats, while assemblage G only infects rodents [18]. Restriction fragment length polymorphism of G. lamblia positive samples, revealed assemblage A in all of the *Mya arenaria* specimens (Figure 2A and Table 4) as evidenced by the expected fragments of 201, 150, 110, and 50 bp. Sequence analysis of the PCR fragment confirmed that they belonged to sub-assemblage AII previously shown to be associated with humans and cats [20]. Likewise, assemblage A was the only genotype observed in the *Crassostrea virginica* positive samples (Figure 2B and Table 4). Sequence analysis revealed *G. lamblia* of the sub-assemblage AI was revealed in all the oyster samples. Sub-assemblage AI is found in cats and humans [20]. We were able to assess the genotype of two of the *G. demissa G. lamblia*-positive specimens (2/3) and found that one specimen belonged to assemblage C as evidenced by DNA fragments of 194, 150, 102, 50, 15 bp, while the other belonged to assemblage D (200, 194, and 117 bp) (Figure 2C and Table 4). The sequencing data support that finding. We genotyped 16/20 *Mytilis edulis* positive for *G. lamblia* DNA. Eight belonged to sub-assemblage AII, while the remainder belonged to assemblage C (Figure 2D and Table 4). Overall, 68% of the bivalves collected at Orchard beach in the fall of 2014 had the *G. lamblia* sub-assemblage AII genotype, while 36% belonged to assemblage C, and 4% belonged to assemblage D (Table 4). The DNA sequences from all the samples were exact matches to reference genotype.

Although, the four species of bivalves examined in this study were collected within feet of each other, a difference in the prevalence of *C. parvum* and *G. lamblia* assemblages was observed. *G. lamblia* assemblage A was detected in *Mya arenaria*, while assemblages C and D were found in *Geukensia demissa*. Interestingly, these two species of bivalves were within inches of each other at the time of collection. Future work with a larger sample size from each species will assess the differential degree of susceptibility of each bivalve species to *C. parvum* and *G. lamblia* genotypes. The *Crassostrea virginica* specimens were collected at different location on Orchard beach. In contrast to previous survey of *C. parvum* in *Crassostrea virginica* collected from the gulf of Maine, we found that the *Crassostrea virginica* specimens were negative for *C. parvum* DNA, but were positive for *G. lamblia* of the assemblage A genotype at a prevalence of 60% [10]. Although a link between eating raw oysters and infection with intestinal parasites such as *C. parvum* and *G. lamblia* has not been established, the screening of oysters for these parasites have been recommended [8].

Assemblages A and B are connected with human infections, whereas assemblages C and D are dog-specific [20,21,22]. This suggests that humans and dogs might have played a role in the contamination of Orchard beach. Intriguingly, Orchard beach was closed during the month of August 2015 because of fecal contamination from humans [23]. This fits well with our data. Additionally, our data indicate that *Mya arenaria* is an excellent bio-indicator species for detection of *C. parvum* and *G. lamblia* in aquatic environment. *Mya arenaria* might be more suitable bivalve species for the screening of human intestinal parasites than the commonly used *Mytilis edulis*. In the future, we will assess the effect of seasonal variation as well as size and age of the bivalve on parasite prevalence.

## 4. Conclusions

We have demonstrated the presence of *C. parvum* and *G. lamblia* DNA in tissues of four bivalve species collected at Orchard beach, NY in the fall of 2014. Our study is the first report of these protozoan human intestinal parasites in bivalves from both Orchard Beach and New York City.

These results are relevant to public health because some mussels are often consumed raw and Orchard beach is well frequented during the warm summer months.

## Figures and Tables

**Table 1:** Prevalence of human intestinal parasites in bivalves from Orchard beach, N.Y.

Mollusks	*C. parvum*	*G. lamblia*	*T. gondii*
*Mya arenaria*	(4/8) 50%	(3/8) 37.5%	0%
*Geukensia demissa*	(5/44) 11%	(2/44) 4.5%	0%
*Crassostrea virginica*	(0/10) 0%	(6/10) 60%	0%
*Mytilis edulis*	(1/97) 1%	(20/94) 20.6%	0%
Total	(10/159) 6.3%	(32/159) 20.1%	0%

**Table 2:** *G. lamblia* tissue distribution prevalence in bivalves from Orchard beach, N.Y.

Mollusks	Foot	Mantle	Digestive Gland	Gills	Siphon	Abductor Muscle
*Mya arenaria*	(1/8) 12.5%	(1/8) 12.5%	0	0	(1/8) 12.5%	ND
*Geukensia demissa*	(1/44) 2.2%	0	0	(2/44) 4.5%	ND	ND
*Mytilis edulis*	(3/97) 3.1%	(2/97) 2.1%	(9/97) 9.3%	(8/97) 8.3%	ND	ND
*Crassostrea virginica*	ND	(3/10) 30%	(4/10) 40%	(2/10) 20%	ND	(3/10) 30%
Total	(5/159) 3.2%	(6/159) 3.8%	(13/159) 8.2%	(12/159) 7.5%		

ND: Not Determined.

**Table 3:** *C. parvum* tissue distribution prevalence in bivalves from Orchard beach, N.Y.

Mollusks	Foot	Mantle	Digestive Gland	Gills	Siphon	Abductor Muscle
*Mya arenaria*	(2/8) 25%	0	(2/8) 25%	0	(1/8) 12.5%	ND
*Geukensia demissa*	(7/44) 16%	(3/44) 6.8%	(3/44) 6.8%	(3/44) 6.8%	ND	ND
*Mytilis edulis*	(1/97) 1%	(2/97) 2.1%	(9/97) 9.3%	(1/97) 1%	ND	ND
*Crassostrea virginica*	0	0	0	0	0	0
Total	(10/159) 6.3%	(5/159) 3.1%	(14/159) 8.8%	(12/159) 7.5%	(/159) 0.6%	

ND: Not Determined.

**Table 4:** *G. lamblia* genotype distribution in bivalves from Orchard beach, N.Y.

Mollusks	Assemblage A	Assemblage B	Assemblage C	Assemblage D	Assemblage E	Assemblage F
*Mya arenaria*	(3/3) 100% Sub-assemblage AII	-	-	-	-	-
*Geukensia demissa*	-	-	(1/2) 50%	(1/2) 50%	-	-
*Mytilis edulis*	(8/14) 57.1% Sub-assemblage AII	-	(8/14) 57.1%	-	-	-
*Crassostrea virginica*	(6/6) 100%	-	-	-	-	-
